# Antibiotics should not be used for back/leg pain

**DOI:** 10.1080/17453674.2020.1871190

**Published:** 2021-01-19

**Authors:** Peter Fritzell, Olle Hägg, Tomas Bergström, Bodil Jönsson, Siv G E Andersson, Mikael Skorpil, Peter Muhareb Udby, Mikkel Andersen

**Affiliations:** a RKC Centre for Spine Surgery in Stockholm, Sweden;; b Spine Center Göteborg, Västra Frölunda, Sweden;; c Department of Infectious Diseases, Institute of Biomedicine, University of Gothenburg, Sweden;; d Sahlgrenska University Hospital, Göteborg, Sweden;; e Department of Cell and Molecular Biology, Biomedical, Center, Uppsala University, Sweden;; f Department of Neuroradiology, Karolinska University, Hospital, Stockholm, Sweden;; g Spine Unit, Ortopaedkirurgisk Afdeling, Sjaellands, Universitetshospital, Køge, Denmark;; h Spine Center of Southern Denmark, Lillebaelt Hospital, Middelfart, Denmark

Acta Orthop 2021; 92 (1): 1–3. DOI 10.1080/17453674.2020.1855561

*Sir,—*It is rather depressing to read the article addressing the topic above in Acta Orthopaedica (Fritzell et al. 2021). By using the definitive title:” Antibiotics should not be used to treat back pain” supported by a highly selective and extremely limited reference list, Fritzell et al. attempt to shut down the low virulent infection hypothesis leading to Modic changes (MC) and chronic low back pain (Fritzell et al. 2021). Readers have to plow through a long and basically irrelevant introduction (Albert et al. 2008, Fritzell et al. 2021) prior to encountering the article’s point of contention. Are antibiotics effective for the treatment of patients with MCs?

1. The authors’ own publication, in which circa 50% of a child cohort complete a questionnaire 13 years after inclusion is likely just an abstraction for most readers as regards making the case for or against the usage of antibiotic treatment for patients with back pain.

2. Fritzell et al. (2019) also refer to their own biopsy article in which they found no evidence of bacteria in the disc material from several patients and in which they conclude that if bacteria were found in an individual patient that this would be due to a contamination process due to the biopsy itself. Many studies (20+) have disproven the contamination hypothesis during the years (Capoor et al. 2019, Manniche and O’Neill 2019. Pradip et al. 2020). And by using fluorescence in situ hybridization microscopy *C. acnes* bacteria can be seen in aggregates and biofilms in human disc material which has initiated a local inflammatory response (Capoor et al. 2017, Ohrt-Nissen et al. 2018). Several leading experts in this field have criticized the results of this study and point to several methodological problems as the reason for their lack of bacterial identification (Capoor et al. 2017).

The interesting considerations in the article are hidden in the last 20 lines (Fritzell et al. 2021). Fritzell describes how a controversial RCT from Bråten et al. (2019) tested whether it was possible to reproduce the same large effect with antibiotic treatment on patients with MCs as the Danish trial (Albert et al. 2013). The Norwegian trial concluded that they did not find a “significant” effect (Bråten et al. 2019). Bråten et al. chose – despite reviewer objections (BMJ 2019) – to mix results for MC type 1 with MC type 2 in their analyses. This is the equivalent of mixing cold and warm water!

The Tables in the Bråten et al. article’s supplementary appendix (2019) told a different story when data for patients with MC1 was presented distinctly from patients with MC2. Data for MC1 patients demonstrated a statistically significant difference and meaningful improvement rarely seen in RCTs involving supervised exercise, manipulation or even spinal surgery for chronic back pain. Patients with MC2 did less well than the placebo group!

Already at the start of the reviewer process and since then a large number of back pain experts have written in different forums about the methodological weaknesses of the study (including the mixing up of data sets) in the Norwegian trial and highlighted the misleading conclusions based upon mixed MC1 and 2 patients (BMJ 2019, Albert 2019, Creaney 2019, Fairbanks 2019, Joffe 2019, Lambert 2019).

The Norwegian authors have recently published a new sub-group analysis of their RCT (Kristoffersen et al. 2020). The conclusions have been significantly modified. A substantial subgroup of patients with MC1 and oedema seen on STIR sequences demonstrated a large difference between the actively treated group and the placebo group as measured by the Roland Morris Disability Questionnaire (RMDQ), the primary outcome measure; –5.1 RMDQ points; 95% CI –8.2 to –1.9; p = 0.008). The clinical improvements were already seen at 3 months and were consistent at 1-year follow-up which showed that 27% of the actively treated group experienced improvements of more than 75%! The Number Needed to Treat (NNT) in this subgroup was 3.1.

The overall conclusion can be that the disc low grade infection hypothesis is a most interesting area of research. This patient group which suffers from longstanding and severe back pain worldwide is deserving of more than simplistic attempts to block further research in this space.

*© 2021 The Author(s). Published by Informa UK Limited, trading as Taylor & Francis Group, on behalf of the Nordic Orthopedic Federation. This is an Open Access article distributed under the terms of the Creative Commons Attribution License (* http://creativecommons.org/licenses/by/4.0/*), which permits ­unrestricted use, distribution, and reproduction in any medium, provided the original work is properly cited.*

DOI 10.1080/17453674.2020.1871190

**Claus Manniche**

*Department of Occupational and Environmental Medicine,*

*Odense University Hospital, and Institute of Clinical Research,*

*University of Southern Denmark, Odense, Denmark*

*E-mail:* Claus.Manniche2@rsyd.dk

Conflict of interest: Shares in Persica Pharmaceutical Ltd., London

*Sir,—*Claus Manniche’s comment to our paper (Fritzell et al. 2021) contains several incorrect claims which taken together create the idea that ordinary back pain could be an infection and the remedy antibiotics.

### Misconception 1: Modic Type 1 change (MC1) on MRI is a cause of chronic low back pain

The implication of Modic changes has engaged spine surgeons and radiologists for more than 30 years (Modic et al. 2008a and b). The conclusion of the Danish long-term follow-up (Udby et al. 2019) concurs with 2 recent reviews stating that “There is no conclusive evidence on the causative role of MC in chronic low back pain (LBP) or any influence on the long-term outcome in patients with LBP or lumbar disc herniations” (Viswanathan et al. 2020) and “the association between MCs and LBP-related outcomes are inconsistent” (Herlin et al. 2019).

### Misconception 2: Infection of the intervertebral disc with Cutibacterium acnes (C. acnes) in cases of MC1 is confirmed by “biofilm” and “inflammatory response”

Several studies have reported this bacterial finding as Manniche states.

*C. acnes* is a commensal of the skin flora with the potential to contaminate sampling procedures. That is what the Swedish study (Fritzell et al. 2019) produced evidence of. Manniche claims that the presence of “aggregates and biofilm” and “local inflammatory response” disproves the evidence. First, aggregates and biofilm are not specific of a disc infection. They can occur in the skin and hair follicles (Jahns et al. 2012). So, a contamination may be in the appearance of bacteria, aggregated bacteria or with a biofilm. The presence of biofilm solely demonstrates that *C. acnes* is capable of this presentation. Biofilm appears to be more related to phylotype of the bacteria than to site or tissue (Kuehnast et al. 2018). Second, the presence of inflammatory response is what you would expect in a case of MC1, since the implication of this phenomenon usually is considered to be just that – an inflammation. If the MRI demonstrates an inflammation above and/or below the degenerated disc, an inflammation in the disc itself is quite feasible. All in all, inflammation is to be expected in MC1. Biofilm is an optional presentation of *C. acnes.*

### Misconception 3: The Norwegian AIM-study (Bråten et al. 2019) obscures the superior effect of antibiotic treatment by presenting the combined effect of MC1 and MC2

This is wrong. In fact, the analyses of the combined group and the MC1 group separately, both demonstrate statistically significant effects in favor of antibiotic treatment. With the outcome measure RMDQ (Roland and Morris 1983, Ogura et al. 2019) the difference in favor of antibiotic treatment is 1.6 units (p = 0.04) in the combined group. In the MC1 group the difference in favor of antibiotics is 2.3 units (p = 0.02). The problem is that the minimal clinically important difference (MCID) of RMDQ is 4–5 units (Maughan and Lewis 2010, Ogura et al. 2019) and the measurement error makes it unable to detect a change smaller than 4–8 units (Stratford et al. 1996, Grotle et al. 2003, Chiarotto et al. 2016). So, a difference of 1.6 or 2.3 is a non-value or a “nonsense value” when it comes to clinical practice. It is like trying to measure a millimeter difference with a centimeter measuring stick. One should not be dazzled by p-values without clinical relevance. We note that those who questioned the conclusion of the AIM-study were nearly all shareholders in Persica, a pharmaceutical company founded to promote the use of antibiotics in back pain.

### *^Misconception 4: A recent subgroup analysis in the Norwegian AIM-study (^*Kristoffersen et al. 2020*^) supports the idea of a low-grade disc infection^*

As the authors themselves conclude, there are several reasons of concern in the interpretation of the analyses. A large number of tests result in a probability of false positive results (Milojevic et al. 2020). They find a difference, exceeding the MCID, in favor of antibiotics for a post-hoc constructed variable constituting 22% of the original study population for one of three outcome measures (RMDQ). Neither back pain (NRS) nor function (Oswestry Disability Index) show clinically relevant or statistically significant differences. This study does not give support to the idea of low-grade disc infection. It regenerates a hypothesis already unsubstantiated and questioned.

So far, the only study that claims the effect of antibiotic treatment of MC1 is published by Manniche himself et al. (Albert et al. 2013). It is a study with a strange and unexplained asymmetrical randomization procedure and a dose response construction which is not reported.

***Conclusion:*** MC1 is not a condition, a disorder, or a disease.It is a finding on MRI. Present scientific evidence does not support prescription of antibiotics for that.
